# Inter-joint coordination with and without dopaminergic medication in Parkinson’s disease: a case-control study

**DOI:** 10.1186/s12984-024-01416-8

**Published:** 2024-07-13

**Authors:** Karolina Saegner, Robbin Romijnders, Clint Hansen, Jana Holder, Elke Warmerdam, Walter Maetzler

**Affiliations:** 1 Department of Neurology, University Hospital Schleswig-Holstein, Campus Kiel and Kiel University, Arnold-Heller Str. 3, Kiel, 24105 Germany; 2 Department of Sport and Exercise Science, University of Salzburg, Salzburg, Austria; 3 Werner Siemens-Endowed Chair for Innovative Implant Development (Fracture Healing), Saarland University, Homburg, 66421 Germany

**Keywords:** Cyclogram, Gait, Kinematics, Motion capture, Neurogeriatrics, Statistical parametric mapping

## Abstract

**Background:**

How the joints exactly move and interact and how this reflects PD-related gait abnormalities and the response to dopaminergic treatment is poorly understood. A detailed understanding of these kinematics can inform clinical management and treatment decisions. The aim of the study was to investigate the influence of different gait speeds and medication on/off conditions on inter-joint coordination, as well as kinematic differences throughout the whole gait cycle in well characterized pwPD.

**Methods:**

29 controls and 29 PD patients during medication on, 8 of them also during medication off walked a straight walking path in slow, preferred and fast walking speeds. Gait data was collected using optical motion capture system. Kinematics of the hip and knee and coordinated hip-knee kinematics were evaluated using Statistical Parametric Mapping (SPM) and cyclograms (angle-angle plots). Values derived from cyclograms were compared using repeated-measures ANOVA for within group, and ttest for between group comparisons.

**Results:**

PD gait differed from controls mainly by lower knee range of motion (ROM). Adaptation to gait speed in PD was mainly achieved by increasing hip ROM. Regularity of gait was worse in PD but only during preferred speed. The ratios of different speed cyclograms were smaller in the PD groups. SPM analyses revealed that PD participants had smaller hip and knee angles during the swing phase, and PD participants reached peak hip flexion later than controls. Withdrawal of medication showed an exacerbation of only a few parameters.

**Conclusions:**

Our findings demonstrate the potential of granular kinematic analyses, including > 1 joint, for disease and treatment monitoring in PD. Our approach can be extended to further mobility-limiting conditions and other joint combinations.

**Trial registration:**

The study is registered in the German Clinical Trials Register (DRKS00022998, registered on 04 Sep 2020).

**Supplementary Information:**

The online version contains supplementary material available at 10.1186/s12984-024-01416-8.

## Background

Bipedal locomotion is a fundamental component of daily life. It is a cyclical movement of limbs, controlled by the central nervous system, during which different body segments are interacting with each other to produce periodic movements [1]. Neurological changes due to aging or neurodegenerative diseases, particularly Parkinson’s disease (PD), can severely impair gait function [1]. PD gait often has a high degree of variability and asymmetry [2, 3], resulting in disturbed walking and instable posture [4], and leading to falling in 50–70% of those affected [5, 6]. Dopaminergic treatment can positively influence these gait deficits, although full recovery in all gait-related aspects is most often not possible [7, 8].

Kinematics describe human motion with regards to joint angles, position, velocity and acceleration [9]. It has already been shown that kinematic aspects of body segments get worse when PD progresses [10] and during dopaminergic off phases [11]. A study investigating kinematics and changes in coordination and its variability has found that the lower limb segment coordination variability is increasing with decreasing functional performance of the lower limb (which is often due to increased disease severity) [12]. Another study revealed that dopaminergic medication improved symmetry between left and right feet during natural walking in persons with PD (pwPD) [11].

Evaluating more than one joint in a person at once allows comparison of their respective kinematics, i.e., inter-joint coordination. Inter-joint coordination is defined as a synchronized movement control of multiple joints, e.g. during a stride [13, 14]. For proper execution of the activities of daily living, an adequate inter-joint coordination must be provided, which can account for different walking speeds, terrains and trajectories, and adapt to changes immediately [15], and can guarantee for a stable, smooth and economic walking [15, 16].

Previously, many studies investigating gait variability have used discrete measurements, e.g., stride length and time [17–20] and derived metrics like coefficient of variation [20, 21]. Only a few studies [12, 22, 23] have used kinematics, especially cyclograms (angle-angle) plots, to quantify inter-joint coordination. Cyclograms allow to look at the spatial and temporal relationship between different body segments or limbs throughout, e.g. a walking cycle [11]. Furthermore, these studies have used statistical analysis on discrete variables obtained from measuring gait, potentially missing important differences occurring throughout the entire gait cycle [24]. To investigate the differences in joint angles during different phases of the gait cycle, statistical parametric mapping (SPM) can be used [24–28], providing insights into kinematic differences between the groups or conditions, e.g. for the whole gait cycle. To the best of our knowledge, none of the aforementioned studies investigated the influence of different gait speeds and medication on/off conditions on inter-joint coordination, as well as kinematic differences throughout the whole gait cycle in well characterized pwPD. As these conditions are highly relevant for informed treatment decisions in PD, we set out to investigate the changes of hip-knee coordination with different walking speeds in pwPD with and without dopaminergic medication using a prospective case-control design. We chose to focus on hip-knee coordination because these are two of the three main joints involved in walking and are significantly affected by PD [29–31].

## Methods

### Participants, in-and exclusion criteria

Participants were included into the study if they were 18 years and older and were able to walk independently without using walking aid. Exclusion criteria were < 15 score in Montreal Cognitive Assessment and other movement disorders that affect mobility performance, as judged by a movement disorder specialist (WM). For detailed inclusion and exclusion criteria we refer to [32]. A total of 58 participants took part in this study. Twenty-nine pwPD (11 females) completed the assessment in the self-perceived best dopaminergic on medication, 30–120 min after intake of levodopa. Eight of them (2 females) also completed the assessment during the medication-off phase (no dopaminergic medication for at least 12 h before the assessment). The control group consisted of 29 healthy adults (14 females) with no mobility-limiting comorbidities as judged by a movement disorder specialist (WM). Demographics and clinical parameters are presented in Table 1.

**Table 1:** Demographic and clinical parameters of the participating groups

Group	HC	PD (on)	PD (off)
Total *N* (males/females)	29 (15/14)	29 (18/11)	8 (6/2)
Age (years)	70 (37–81)	66 (48–85)	62 (48–80)
Height (cm)	174 (151–199)	173 (154–195)	177 (169–195)
Weight (kg)	79 (52–112)	82 (38–124)	85 (59–124)
BMI (kg/m^2^)	25 (18-39)	27 (16-35)	28 (21-35)
Disease duration (years)	-	7 (1-25) 6 (2-18)*	6 (2-18)
Hoehn & Yahr	-	3 (1-4) 2.5 (1-3)*	2.75 (1-3)
Medication dose (LEDD)	-	626 (266–1380)	-
MDS-UPDRS III	5 (0–17)	24 (3–80)	
		23 (11-47)*	28 (13-45)

*only pwPD measured both on and off medication. Values are displayed as median (range). BMI – Body Mass Index; HC – healthy controls; MDS-UPDRS-III – Motor part of the Movement Disorder Society Unified Parkinson’s Disease Rating Scale; PD (on) – PD medication on group; PD (off) – PD medication off group

### Equipment

Participants were measured with a twelve-camera optical motion capture system (Qualisys AB, Göteborg, Sweden) recording full-body movements with 200 Hz. The data from 20 markers, placed on lower limbs, were used in this study. Full details on experimental set up can be found in Additional material A-I and [32].

### Motor assessment

Participants walked straight along a 5 m long and 1 m wide walkway performing three walking trials, namely at preferred (“Please walk at your normal walking speed”), slow (“Please walk half of your normal walking speed”), and fast (“Please walk as fast as possible, without running, falling or feeling unsafe”) speeds. The start and end of the track were marked by cones with reflective markers. Participants were asked to start approximately 2 m in front of the start cones and to finish approximately 2 m behind the end cones to ensure that steady state gait was measured within the 5 m of gait assessment and any artificial variation in gait, occurring during gait initiation and termination [33, 34] is excluded.

### Marker data pre-processing

Marker data was prepared in a format that inherits from the Brain Imaging Data Structure (BIDS) [35], and were loaded into MATLAB (Matlab R2017a; The MathWorks Inc., Natick, MA, USA). Not all participants completed all trials, thus 12 trials were missing and a total of 186 trials with corresponding static data to obtain the reference position could be uploaded.

Using a custom written script, data missing at least one of the markers for the entire trial was excluded from the analysis (*N* = 9). Gaps in the marker trajectories for the remaining 177 trials were filled based on marker correlations [36]. Ten trials were subsequently excluded due to suboptimal prediction (see A-II, Fig. A1 and A2). Details regarding data filtering can be found in Additional material A-III.

Gait events, i.e., initial and final contacts, as well as the timing of crossing the start and end line, were previously detected by [37] and were used in this study for the remaining 167 walking trials.

### Joint angle extraction

The joint angles were extracted using a custom-written Python script (available at [38]). Detailed explanation of joint angle extraction from motion capture data can be found in the Additional material (A-III).

### Data extraction and presentation

The rotation angles around the three biomechanical axes were represented graphically. Subsequently, seven trials were excluded based on predefined criteria for joint angles (details can be found in Additional material A-IV). Therefore, 160 trials were available for the analysis of the rotation around the medio-lateral axis (representing the movement in the sagittal plane) between the start and stop markings.

Further pre-processing steps included determining the gait cycles, their linear interpolation and mean-centering of the data. Detailed explanation can be found in the Additional material (A-IV).

#### Data-derived variables

The following variables were calculated:

**Range of motion (ROM)** is defined here as the average angular range of each joint (hip and knee) during a cycle in the sagittal plane.

**Angular coefficient of correspondence (ACC)** quantifies the cyclogram variability within the subject based on consecutive differences of hip-knee angular relationship. The values range from 0 to 1, where 0 indicates no consistency and 1 indicates perfect consistency in the hip-knee relationship over multiple cycles.

The **sum of squared distances (SSD)** quantifies the cyclogram variability based on consecutive differences of cyclogram shapes. This variable allows to calculate the differences in cyclograms within participants (between different cycles), or between the average group cyclograms obtained from different walking conditions. Higher values indicate higher variability.

The **ratio of minimum points to ratio of maximum points** describes the adaptation behavior during the gait cycle between different walking speeds, where low values indicate late, and high values early adaptation during the gait cycle (see also discussion).

Detailed explanation on data-derived variables can be found in the Additional material (A-V).

### Statistics

Statistical analysis was done using JASP (JASP Team, version 0.18, University of Amsterdam, The Netherlands). Shapiro-Wilk test was used to test if the data is normally distributed. The characteristics of all groups are presented as median and range. For normally distributed data, outliers were removed by calculating the Z score for each variable and removing those 3 standard deviations above or below the mean. For non-normally distributed data, outliers were removed by calculating the interquartile range and removing those values which were 1.5 times greater or less than the interquartile range.

To compare gender distribution between the groups, a chi-squared test was used. To compare all the other variables between the groups, t-test and Mann-Whitney U tests were used for normally and non-normally distributed data, respectively. To compare the variables between different walking speeds (within the groups), either a repeated measures ANOVA or a Friedman’s test were used. In case of normally distributed data and significant main effects, Bonferroni post hoc comparisons were used. In case of non-normally distributed data and significant main effects, Conover’s post hoc test was used. For comparing the values between PD medication on and PD medication off groups, Wilcoxon signed-rank test was used. Significance level was set to 0.05.

Pre-processed time-series data of the entire gait cycle (pre-processing details can be found in Additional material A-III) were analyzed using SPM (SPM1d Python version 0.4.18, available at [39]). Data distribution was checked using Shapiro-Wilk test (spm1d.stats.normality.shapirowilk). An independent samples t-test (spm1d.stats.ttest2) was used to compare PD medication on group against controls. A paired samples t-test (spm1d.stats.ttest_paired) was used to compare PD medication on and PD medication off groups. Significance level was set to 0.05.

## Results

After removing the outliers (*n* = 2), 158 trials (fast walking *N* = 50, preferred walking *N* = 55, slow walking *N* = 53) were analyzed. The groups (controls, PD medication on group, PD medication off group) did not significantly differ in gender distribution, age, height, weight or body mass index (BMI) (*p* > .05). The MDS-UPDRS III scores differed between the control and PD groups and showed a trend towards significance between the PD medication on group and the PD medication off group (higher in the latter, *p* = .06). Details are shown in Table 1.

### PD gait differs from normal gait mainly by a reduced knee ROM, and adaptation to gait speed in PD is mainly driven by an increase of hip ROM

The comparison of the ROM of the cyclograms between walking speeds and groups showed the following results within groups: in the control group, hip ROM differed between all walking conditions, being greatest in the fast walking condition and smallest in the slow walking condition; knee ROM was smaller in the slow walking condition, compared to both fast and preferred walking condition. In the PD medication on group, hip ROM was smaller in the slow walking condition, compared to both fast and preferred walking conditions; the same was observed for knee ROM. In the PD medication off group, both hip and knee ROM in the slow walking condition were smaller compared to the hip and knee ROM in the fast and preferred walking condition.

Between groups, hip ROM was greater in the control group than in the PD medication on and off groups in the fast walking condition and greater than in the PD medication off group in the preferred walking condition; knee ROM was greater in the control group than in the PD medication on and off groups in all walking conditions. Neither hip nor knee ROM differed between the PD medication on and off groups. Details are shown in Table 2.

**Table 2:** Kinematic hip and knee parameters and ratios of the cyclograms

	Controls (*N* = 29)			PD medication on (*N* = 29)					
				PD medication on (*N* = 8 that were also measured in medication off)			PD medication off (*N* = 8)		
**Walking cond.**	**Fast**	**Preferred**	**Slow**	**Fast**	**Preferred**	**Slow**	**Fast**	**Preferred**	**Slow**
ROM hip [degree]	46 (35-56)	41 (20-48)**	33 (20-43)**^††^	41 (21–58)^×^	36 (20-49)	30 (16-44)**^†^			
				46 (21-50)	39 (20-43)	34 (17-37)*	37 (19-45)^××^	36 (17-41)^×^	31 (16-37)*^†^
ROM knee [degree]	64 (53–70)	62 (48–68)	59 (42–70)**^††^	55 (41–70)^××^	54 (41–69)^××^	50 (35–65)**^††××^			
				56 (41–70)	55 (41–69)	53 (35–62)	52 (35–58)^××^	55 (36–59)^××^	45 (33–64)*^†××^
ACC	0.98 (0.96-1.00)	0.99 (0.92-1.00)	0.95 (0.76–0.99)**^††^	0.98 (0.93–0.99)	0.97 (0.92–0.99)^××^	0.96 (0.84–0.99)*			
				0.98 (0.96–0.99)	0.98 (0.92–0.99)	0.96 (0.88–0.99)	0.98 (0.94-1.00)	0.98 (0.91–0.99)	0.97 (0.79–0.99)*
SSD	4.3 (2.5–7.9)	3.3 (1.8–6.2)*	4.2 (2.9–14.7)^††^	4.6 (2.3–9.7)	4.6 (2.6–6.3)^××^	4.0 (2.7–11.0)			
				4.5 (2.8–6.5)	4.5 (2.6–5.6)	3.7 (2.7–5.3)	3.5 (2.7–4.4)	3.5 (3.1–5.1)	5.0 (2.0–6.0)
**Comparisons**	**Fast vs. Pref**	**Fast vs. Slow**	**Pref vs. Slow**	**Fast vs. Pref**	**Fast vs. Slow**	**Pref vs. Slow**	**Fast vs. Pref**	**Fast vs. Slow**	**Pref vs. Slow**
Ratio of minimum points to ratio of maximum points	1.20 (1.05–1.38)	1.61 (1.19–2.46)	1.33 (1.00-1.78)	1.15 (0.91–1.38)^×^	1.38 (0.97–1.83)^×^	1.19 (1.00-1.45)^×^			
				1.13 (1.08–1.38)	1.33 (1.19–1.83)	1.22 (1.07–1.32)	1.09 (1.04–1.21)^×^	1.25 (1.24–1.32)^×^	1.17 (1.03–1.26)^×^

Range of motion (ROM) of hip and knee joints, angular coefficient of correspondence (ACC), sum of squared distances (SSD) and ratio of minimum points to ratio of maximum points of the cyclograms (median (range)). The values of PD subgroup assessed in medication on and medication off are in the second row for each variable. ^* / **^p < .05 /0.01 against fast walking condition; ^† / ††^*p* < .05 /0.01 against preferred walking condition; ^× / ××^*p* < .05 /0.01 against controls in the same walking condition. Fast – fast walking speed; PD – Parkinson`s disease; Pref – preferred walking speed; Slow – slow walking speed

### Regularity of hip-knee cyclograms is reduced in PD but only during preferred speed

The comparison of ACC and SSD of the cyclograms between walking speeds and groups showed the following results within groups: in the control group, ACC was greater in the fast and preferred walking conditions than in the slow walking condition. In both PD medication on and off groups, ACC was greater in the fast walking condition than in the slow walking condition. The SSD of the preferred walking speed in the control group was smaller than that of the fast and slow walking conditions. No differences in SSD were found between the different walking conditions in either the PD medication on or PD medication off groups.

Between groups, ACC was greater in the control group than in the PD medication on group in the preferred walking condition. The parameter did not differ between the PD medication on and off groups. Comparably, SSD was smaller in the control group than in the PD medication on group in the preferred walking condition. Also, this parameter did not differ between the PD medication on and off groups. Details are shown in Table 2.

### PD is associated with altered adaptation strategies of hip and knee movements to different walking speeds

**Fig. 1:**
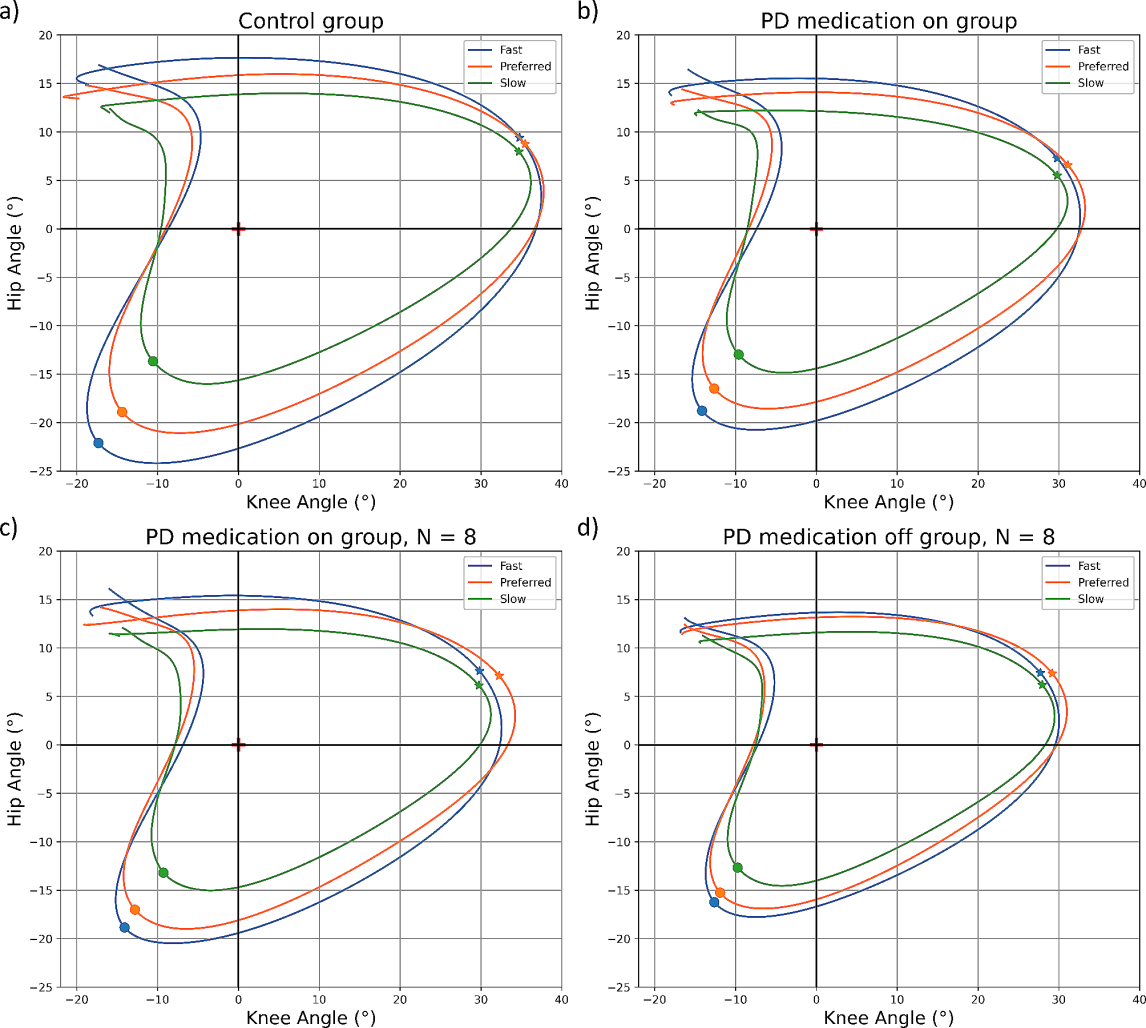
Average cyclograms of the groups Average cyclograms of controls (subplot a), PD medication on (subplot b), PD medication on (only those measured both on and off medication (subplot c)) and PD medication off (subplot d) from fast (blue), preferred (orange) and slow (green) walking speed trials. Red cross (0,0) indicates cyclogram centroids. The circles indicate the minimum, and the stars indicate the maximum cyclogram points. PD – Parkinson’s disease

In order to evaluate the adaptation processes within gait cycles, minimum and maximum cyclogram points were calculated, evaluated where they occur in the gait cycle (identified as % of the gait cycle), and ratios of minimum points to ratios of maximum points of the cyclograms were established. The minimum cyclogram point can be understood as the point where the body transfers its weight from the heel to the forefoot for forward propulsion, just before toe off (circles in Fig. 1). The maximum cyclogram point can be understood as the point of maximum flexion in both joints combined, when the leg is in the air before the ground contact (stars in Fig. 1). For all groups and all speeds, the minimum cyclogram point appeared in 46 ± 2% of the gait cycle, which is about 2/3 into stance phase [40]; the maximum point appeared in 76 ± 1% of the gait cycle, which is almost the middle of the swing phase [40]. Ratios of minimum points to ratios of maximum points of the cyclograms were greater in the control group (Fig. 1a) than in the PD medication on (Fig. 1b) and off (Fig. 1d) groups in all three comparisons (fast versus preferred, fast versus slow, and preferred versus slow). No differences were found between the PD medication on (Fig. 1c) and off (Fig. 1d) groups. Details are shown in Table 2.

### PD is associated with decreased knee kinematics in the swing phase during different walking speeds

**Fig. 2:**
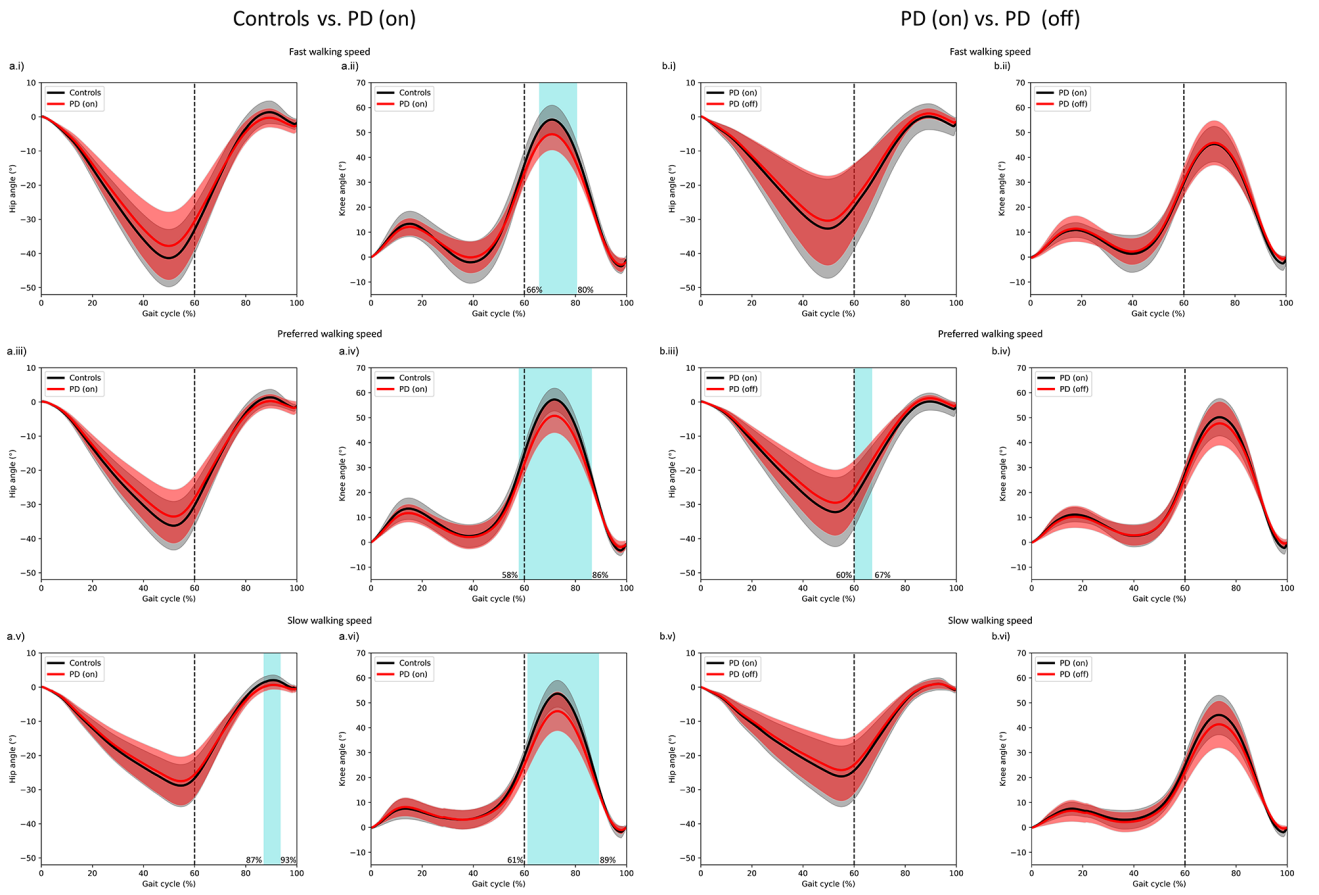
SPM results for temporally aligned hip and knee angles SPM results for temporally aligned hip (first and third columns) and knee (second and fourth columns) angles in fast (first row), preferred (second row) and slow (third row) walking speeds. In subplots a, controls (mean in black, SD in light grey) are compared to PD medication on group (mean in red, SD in light red). In subplots b, PD medication on (mean in black, SD in light grey) are compared to PD medication off (mean in red, SD in light red). Light blue blocks indicate significantly different parts (*p* < .05) of the gait cycle. The percentages on the left side of the significance block indicate the start, and the percentages of the right indicate the end of significant differences in angles across the gait cycle. Vertical dashed line indicates the change from stand phase to swing phase

To evaluate where in the gait cycle the hip and knee angles differ between PD medication on group and controls, SPM analysis on temporally aligned data was used, allowing a spatial comparison of the gait cycles. Hip angles were larger in the control group than in the PD medication on group in the slow walking condition in the late swing phase of the gait cycle (Fig. 2a.v). Knee angles were larger in the control group than in the PD medication on group in all walking conditions (Fig. 2a.ii, 2a.iv and 2a.vi). All the significant differences appeared during the swing phase of the gait cycle.

### Dopaminergic medication improves the hip kinematics only in preferred walking speed

SPM analysis on temporally aligned data was used to evaluate the differences in the gait cycle between PD medication on and off groups. Hip and knee angles increased when the subjects were walking on medication, however the only significant difference was observed in the hip angles when walking in preferred speed during the early swing phase of the gait cycle (Fig. 2b.iii).

### PD is associated with delayed peak hip flexion

**Fig. 3:**
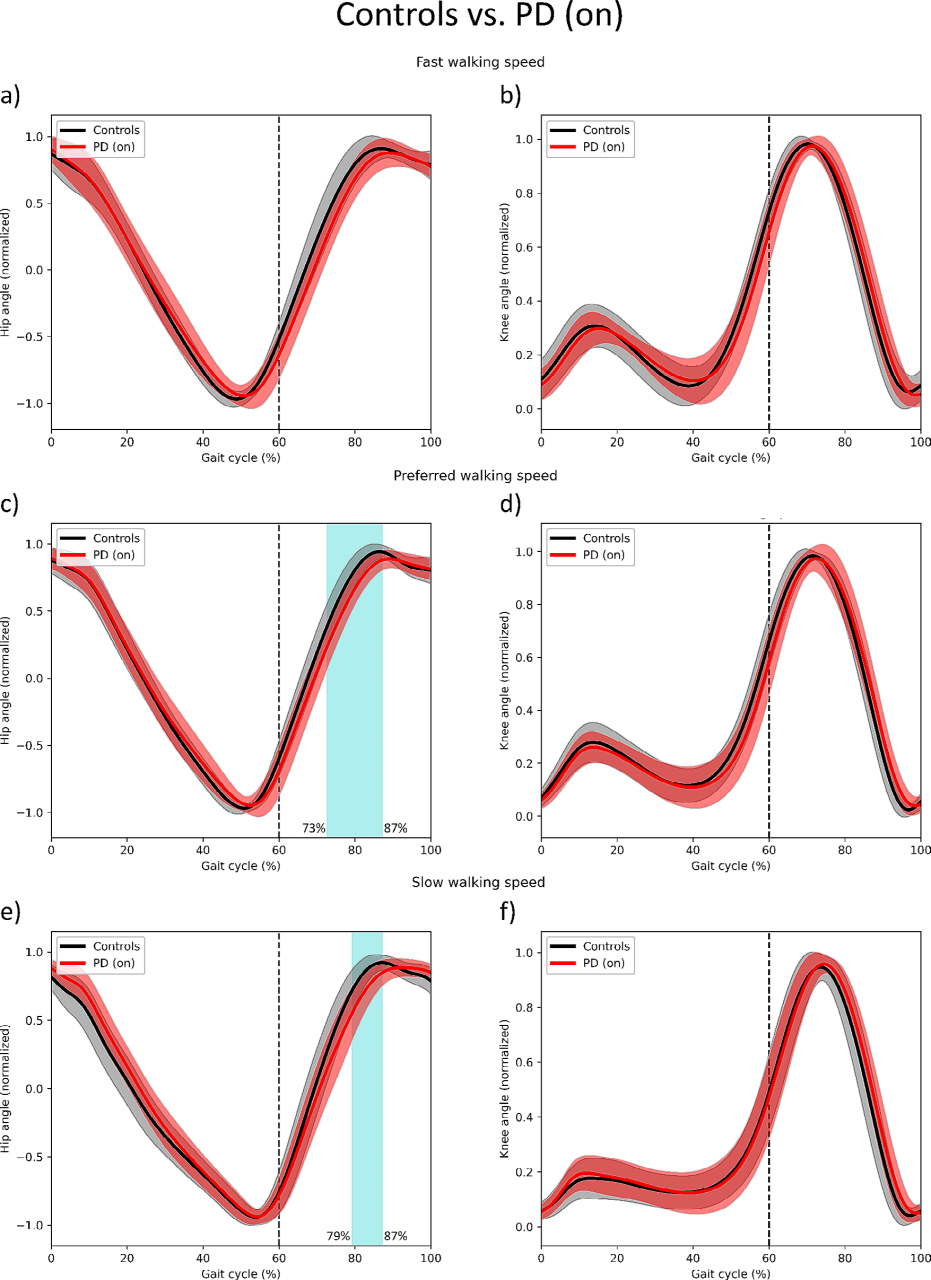
SPM results for spatially aligned hip and knee angles SPM results for spatially aligned hip (first column) and knee (second column) angles in fast (first row), preferred (second row) and slow (third row) walking speeds. Light blue blocks indicate significantly different parts (*p* < .05) of the gait cycle between the controls (mean in black, SD in light grey) and PD medication on group (mean in red, SD in light red). The percentages on the left side of the significance block indicate the start, and the percentages of the right indicate the end of significant differences in angles across the gait cycle. Vertical dashed line indicates change from stand phase to swing phase

To evaluate where in the gait cycle do certain differences in movement between groups appears, SPM analysis on spatially normalized data was used, allowing a temporal comparison of the gait cycles. During the middle of the swing phase, controls reached the peak hip flexion earlier than the PD medication on group in preferred and slow walking speeds (Fig. 3c and e).

There were no temporal differences observed between PD medication on and off groups.

## Discussion

This prospective, observational case-controlled study describes kinematics of the two joints largely involved in walking, namely hip and knee, and their inter-joint coordination during walking and how they are associated with walking speed in pwPD on and off medication. Furthermore, the study includes a detailed comparison of joint movements throughout the gait cycle. These changes were evaluated by the means of cyclograms and comparisons thereof, as well as using SPM and comparing time series of gait cycle between the groups. The ability of these metrics to differentiate between healthy controls and pwPD during on and off medication suggests such analysis as promising intermediate clinical endpoints [41]. These aspects will be discussed with a focus on mechanistic and clinically relevant implications.

First, ROM results suggest that knee movement during walking is (i) particularly affected in PD and (ii) particularly inefficient in adapting to changes in walking speed. This is supported by the SPM results on the temporally aligned knee angles, which showed that pwPD had significantly lower knee angles during a relevant part of the swing phase of the gait cycle (62nd -85th percentile of the gait cycle) for all walking speeds, when compared to controls. One of the symptoms that often occurs in PD is axial rigidity [42]. This should, by definition, affect the hip more than the knee, and therefore many physiotherapy interventions indeed focus on improving hip function. However, our results suggest that interventions that focus on the increase of knee angles during walking may be particularly beneficial in enabling pwPD to better adapt their walking performance to changing environmental demands. Furthermore, we did not observe any significant increases in knee ROM when the participants were walking on medication, compared to their off phase; however, there was a slight improvement due to medication of the hip ROM at the beginning of the swing phase (Fig. 2). This finding is particularly interesting because it implicitly allows the conclusion that dopaminergic medication - from a kinematic point of view - on the one hand cannot reverse the deficit in hip-knee movement during walking (reduction in knee ROM) ‘produced’ by PD in any way (there are no significant ROM differences in knee movement between medication off and medication on phase), and on the other hand the compensation by an increased movement of the hip is quite late in the gait cycle and only very modest (Fig. 2). This finding suggests that PD-induced neurodegeneration of the basal ganglia is too complex to be compensated for by ‘simple’ administration of dopaminergic medication, and could also explain the small effect that dopaminergic medication has on PD-induced gait disorders [7, 43, 44]. Our data also indicate that dopaminergic medication is particularly beneficial for ‘simple’ activities (here under preferred speed), but not for more complex activities [8, 45].

Second, ACC and SSD values showed that gait regularity decreased in all groups with decrease of walking speed from preferred to slow. This was expected from clinical experience and the literature. For example, similar results were observed in a case-controlled study of people with spinal cord injury [23]. Interestingly, the SSD was greater (indicating worse gait regularity) in controls under fast walking speed than under preferred speed, which was not the case in the PD group. In addition, in the PD group, the ACC improved, albeit not significantly, under fast compared to preferred speed. It therefore appears that, in pwPD, walking above the “comfort zone” of gait speed can improve the quality of walking. An improvement in the arousal state during fast walking, which may not be optimally regulated under ‘comfort zone’ conditions in pwPD [46] may be one explanation for this phenomenon.

Third, the comparison of the ratio of minimum points to ratio of maximum points of the cyclograms between gait speeds and groups showed that PD is associated with altered adaptation strategies of hip and knee movements to different walking speeds. The ratio of minimum points can be understood as a surrogate marker for the joint movement in the hip and knee at the end of the stance phase. This is the phase in which the person generates the momentum for the subsequent swing phase. The greater this ratio is, the more dynamic the preparation for the subsequent step. The ratio of maximum points can be understood as a surrogate marker for adaptive / corrective movements in the middle to end of the swing phase of a step. This is a phase in which an adaptation, e.g., to increasing walking speed, appears too late or is no longer efficient. In our view, the ratio of minimum points to ratio of maximum points of the cyclograms is therefore a helpful surrogate marker for a person’s adaptive strategies to increased walking speed. Table 2 shows that controls produce 61% difference in this parameter between fast and slow pace, while pwPD on medication produce only 33%, and pwPD off medication only 25% difference. These data suggest that pwPD develop less momentum in the “production” of the swing phase of a step from the hip and knee, which is then reflected in smaller joint angles throughout the swing phase, as indicated by SPM analysis on temporally aligned data. Confirmation of this hypothesis would entail interesting new specific training options e.g., not only having a treadmill training with changing walking speed [47], but putting particular focus on the late stance phase of the stride.

Fourth, the SPM analysis on spatially normalized data showed that PD is associated with altered hip joint behavior during gait. The PD medication on group reached the peak flexion in the swing phase in preferred and slow speeds later than controls. Furthermore, no differences were observed between medication on and off groups, suggesting again that medication does not play a crucial role in improving gait events, which now refer to the temporal aspects. Usual physiotherapy interventions focus on training gait as a whole, e.g., on a treadmill [48, 49], or by resistance training [50], which has shown positive results in increasing gait velocity and reducing fall risk. However, none of these studies have focused specifically on the swing phase of the stride. Our results suggest that particular attention should be paid to the hip [51], namely flexing the hip faster and reaching the peak flexion of the hip earlier, thus allowing pwPD to increase gait speed and adjust to the surroundings more effectively.

This study has strengths as well as limitations. Strengths are the application of kinematics including two adjacent large joints to a well-defined diseased cohort with and without treatment during different speeds of walking, the inclusion of a control cohort, and the discussion of the results at the interface of movement and clinical science. Results motivate future studies to analyze, e.g., cohorts with other mobility-limiting diseases, the relationship of other than hip and knee joints during movement, and the movement pattern in other than the sagittal plane. Furthermore, the analysis methods could be used on data collected with wearable devices, e.g. inertial measurement units, in daily life [52, 53]. Limitations are the small group size of patients in off medication state, high disease variability, the focus of relatively short bouts of straight walking and no specific procedure for measuring the gait speed.

## Conclusions

In conclusion, this prospective, case-controlled study reports on a potentially highly relevant and, with the advent of new markerless tracking systems, very easily applicable method for assessing movement and, more specifically, kinematic deficits. It reveals distinctive inter-joint coordination patterns in persons with Parkinson’s disease (pwPD) during walking. While axial rigidity traditionally emphasizes hip-focused interventions, our findings advocate for targeted knee-angle improvements to enhance pwPD’s adaptive walking performance. Gait regularity analysis indicates that walking faster than usual may improve gait quality in PD, potentially linked to enhanced arousal states. Furthermore, the SPM analysis highlights delayed peak hip flexion during the swing phase in pwPD, prompting consideration for swing phase-focused training interventions. Dopaminergic medication has no significant influence of these differences, arguing in favor of non-medication treatment of such gait deficits in PD. This study provides valuable insights into gait dynamics in PD by use of an innovative kinematics approach, which can also inform future investigations into further inter-joint movement comparisons and other cohorts.

### Electronic supplementary material

Below is the link to the electronic supplementary material.

Supplementary Material 1

## Data Availability

Part of the data analysed during the current study are available at https://www.mdpi.com/2306-5729/7/10/136. The remaining data contains patient information and are available from the corresponding author on reasonable request. The joint angle extraction code is available at https://github.com/neurogeriatricskiel/MocapJointAngles. It is platform independent, written in Python programming language. Requirements include Python version 3.11 or higher.

## Abbreviations

PD: Parkinson’s Disease
pwPD: Persons with Parkinson’s Disease
SPM: Statistical Parametric Mapping
BIDS: Brain Imaging Data Structure
ROM: Range of Motion
ACC: Angular Coefficient of Correspondence
SSD: Sum of Squared Distances
BMI: Body Mass Index
