# Supplementary material for: Inter-joint coordination with and without dopaminergic medication in Parkinson’s disease: a case-control study
Source: J Neuroeng Rehabil. 2024 Jul 13;21:118. doi: 10.1186/s12984-024-01416-8 (PMC11245779; doi:10.1186/s12984-024-01416-8)
Supplement: Supplementary file 1 — Supplementary Material 1 [file 12984_2024_1416_MOESM1_ESM.docx]

**Additional material**

*A-I. Experimental set-up*

A total of 47 reflective markers (19 mm in diameter) were placed on the body using either adhesive tapes or elastic bands with or without a rigid plate. For calibration purposes, additional eight reflective markers were placed on bony landmarks to estimate the joint positions. For this analysis, data from 20 markers were used (Fig. A1a). Four markers were placed in the area of the anterior and posterior superior iliac spines. Sixteen markers were placed on four rigid plates (four markers per plate, making a marker cluster), which were then attached to the middle of thigs and shanks using elastic bands.

*
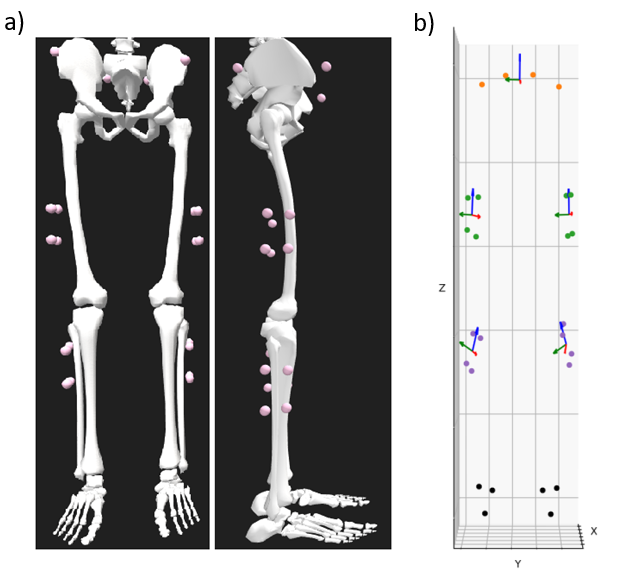
*

Figure A1. Subplot a – placement of the reflective markers. Subplot b – static markers in frontal plane with local coordinate systems. Red, green and blue arrows indicate x, y and z directions of the local coordinate systems, respectively. Orange dots – pelvic markers; Green dots – thigh marker clusters; Purple dots – shank marker clusters; Black dots – feet markers.

*A-II. Marker prediction*

Example plot of poor marker prediction (prediction algorithm from [33]).


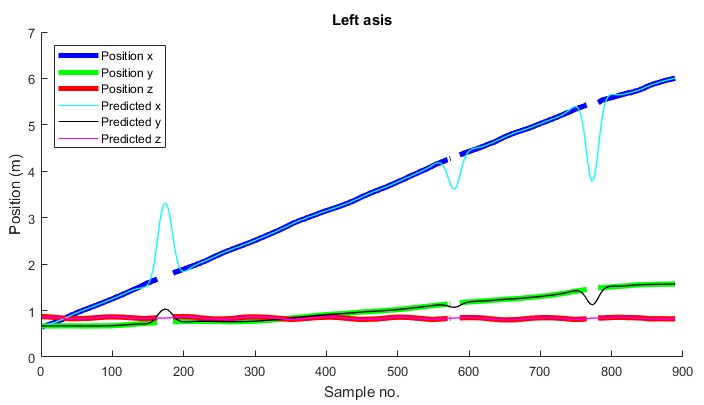


Figure A2. Recorded (bold lines) and predicted (thin lines) left anterior posterior iliac spine (asis) marker positions from motion capture data. X, y and z denote the marker positions in x, y and z directions in the global coordinate system, respectively.


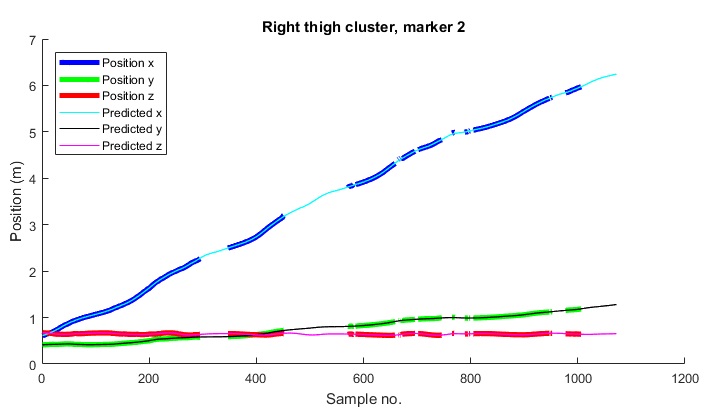
Example plot of acceptable marker prediction (prediction algorithm from [33]).

Figure A3. Recorded (bold lines) and predicted (thin lines) of the second right thigh cluster marker positions from motion capture data. X, y and z denote the marker positions in x, y and z directions in the global coordinate system, respectively.

*A-III. Joint Angle extraction*

First, the marker data was filtered using a 2^nd^ order low-pass Butterworth filter (cut-off frequency = 6 Hz). Then the local coordinate systems were computed from both static and dynamic data for the pelvis and both thighs and shanks (Figure A1b). Then a transformation matrix for the pelvic, thigh and shank markers was calculated. For the static trials, the average of the marker positions was used; for the dynamic trials, the marker positions were multiplied by the transformation matrix for each time stamp. To obtain the position, the transformation matrix of each time stamp from the dynamic data was multiplied by the inverse transformation matrix of the corresponding static data. The left and right Euler angles for the hip rotations around the three axes (vertical, antero-posterior and medio-lateral) were determined by multiplying the inverse pelvic transformation matrix with the left and right thigh transformation matrices; similarly for the knee rotations the inverse left and right thigh transformation matrices were multiplied with the corresponding shank transformation matrices. As the last step, Euler angles were converted to degrees using the following rotation sequence (Equations 1-3):

| $\alpha=arcsin(R[2,1]) * 180/\pi$ | (1) |
| --- | --- |
| $\beta=arctan2(-R[2,0], R[2,2]) * 180/\pi$ | (2) |
| $\gamma=arctan2(-R[0,1], R[1,1]) * 180/\pi$ | (3) |

where α, β and γ represent the rotations around X, Y and Z axes, respectively, and R is the rotation matrix.

*A-IV. Angle data extraction, pre-processing and presentation*

Exclusion criteria for the joint angles obtained during the joint angle extraction from motion capture data were more than 20 and 10 degrees for hip and knee internal/external rotation and more than 10 and 5 degrees for hip and knee abduction/adduction), as these values were deemed outliers and not representative of the intended joint motion.

The gait cycles were determined for the left and right legs by identifying the onset of the first initial contact of one side until the following initial contact of the same side. The hip and knee angles were saved per cycle, allowing to have multiple cycles saved per side (left and right) and per walking speed (slow, preferred, fast) for each participant.

Further pre-processing steps included linear interpolation (500 samples per gait cycle[23]), which was used to even out the number of samples per cycle for all participants and all walking speeds, as well as mean-centering the data for each cycle. Note that mean-centering moves the angles into hyperextension, which appears when the mean is subtracted from all data points. After these pre-processing steps the hip and knee angles were plotted against each other to create cyclograms. Each gait cycle had a corresponding cyclogram with the centroid origin being at (0,0). Example cyclograms are presented in Figures A4 and A5.

The pre-processing for SPM analysis was made in a few steps. First, to align all gait cycles between the participants, the value of the first sample was subtracted from all remaining samples, bringing the cycle start to 0. For spatial comparisons of the gait cycles and to account for differences in walking speed and align the trials temporally, an Elastic Functional Alignment (47) technique was applied (fdasrsf.fdwarp). An example of the raw and aligned data can be found in the Additional material (Fig. A6). For the temporal comparison of the gait cycles, the data from each cycle was normalized to have the values between -1 and 1 for the hip angles, and between 0 and 1 for the knee angles.


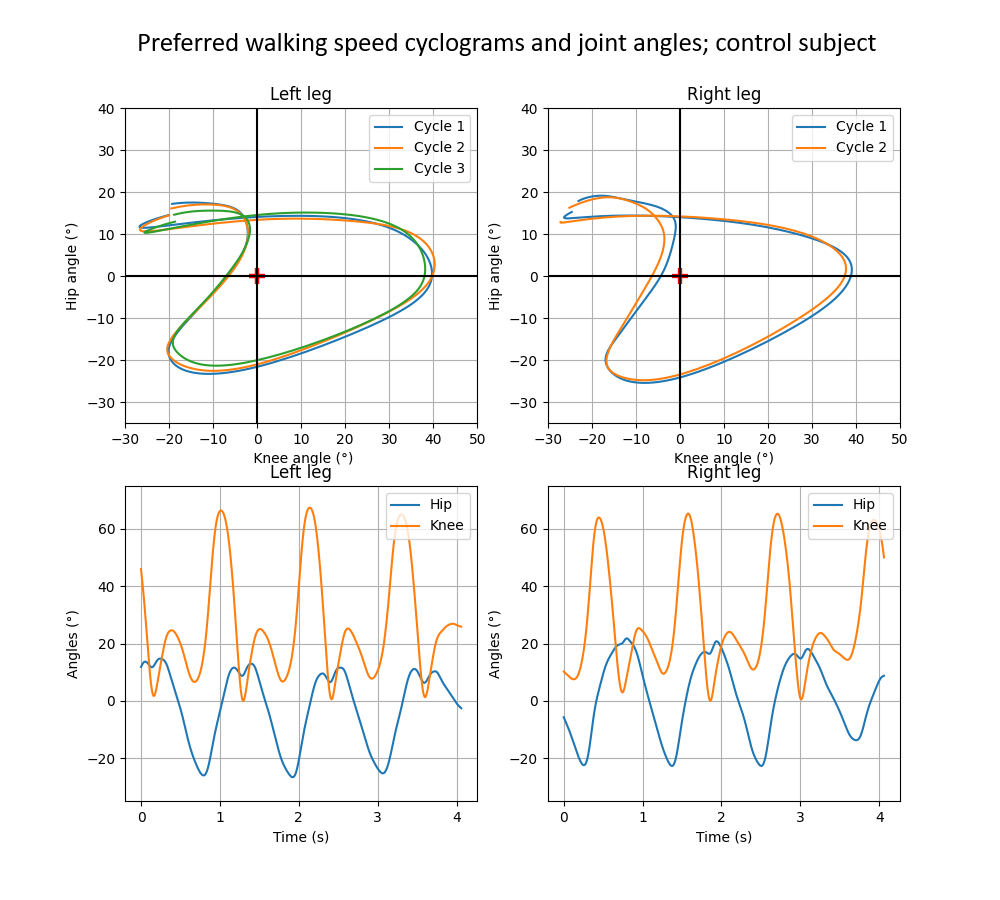

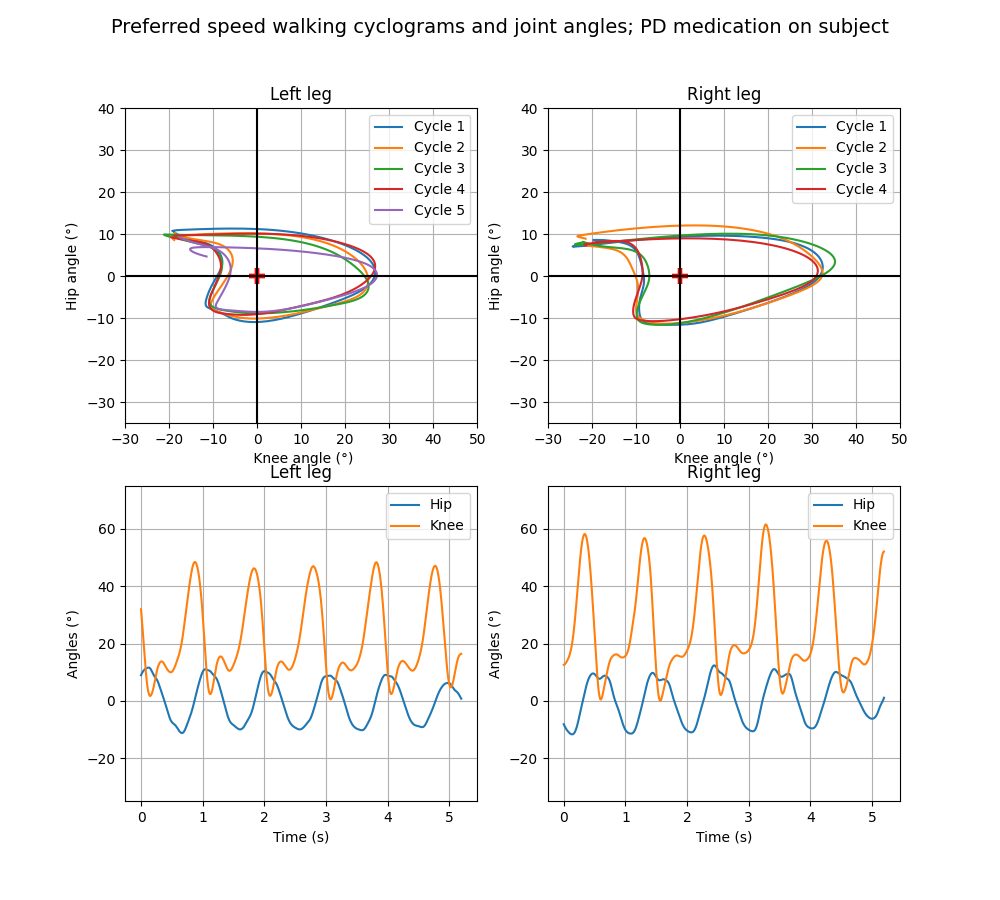


Figure A4. Example cyclograms from a healthy control subject walking in preferred speed. The cyclogram origins are adjusted to have the same origin of (0,0), and are indicated by a red cross. The knee flexion angles are adjusted to have maximum extension of 0°.

Figure A5. Example cyclograms from a PD medication on subject walking in preferred speed. The cyclogram origins are adjusted to have the same origin of (0,0), and are indicated by a red cross. The knee flexion angles are adjusted to have maximum extension of 0°.


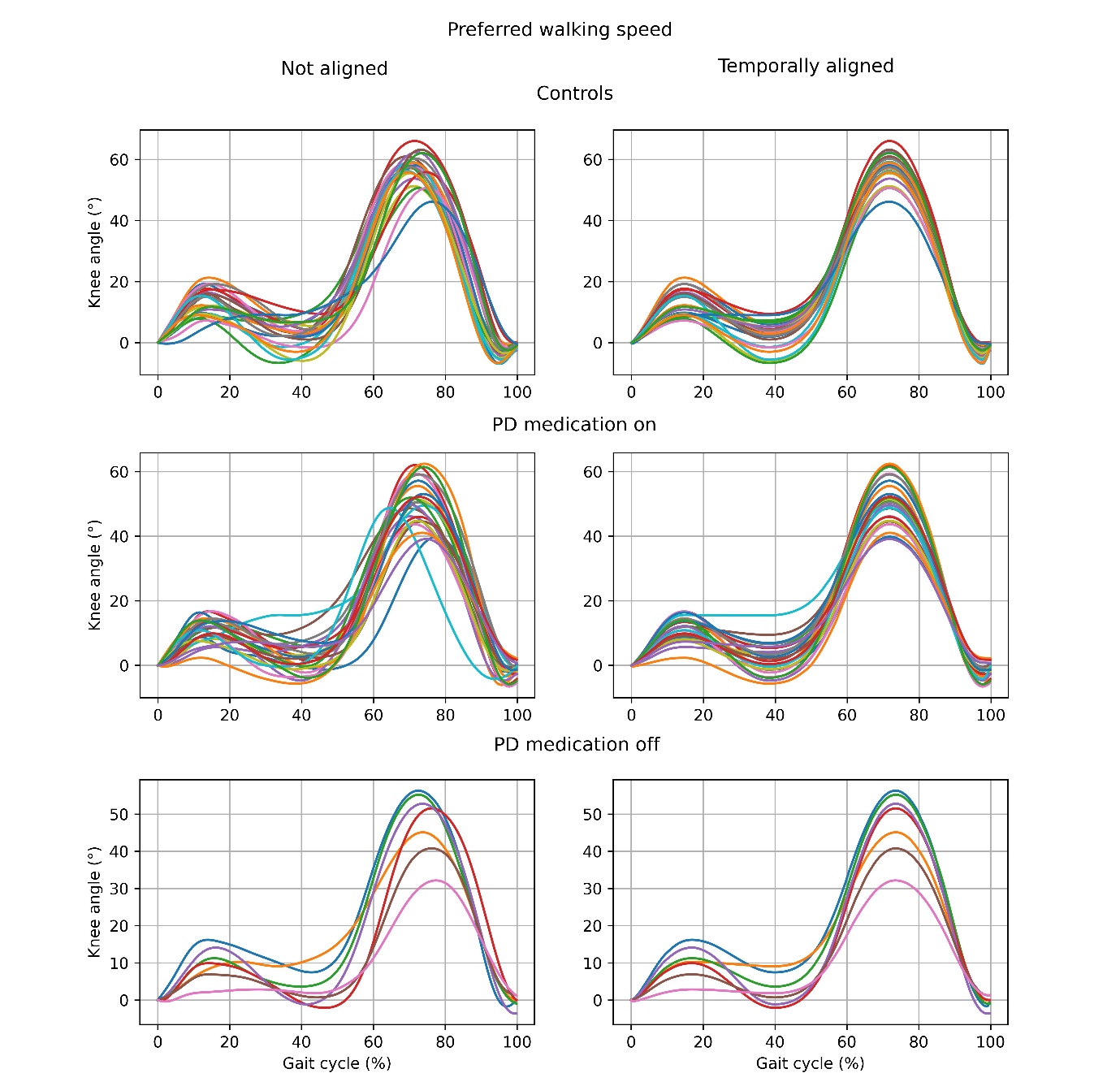


Figure A6. Example of temporal knee angle data alignment for all groups in preferred walking speed.

*A-V.* *Data-derived variables*

**Range of motion (ROM)** is calculated by subtracting the minimum angle value from the maximum angle value for one cycle. ROM for each participant and each walking speed was calculated by taking the mean ROM from all cycles per trial.

**Angular coefficient of correspondence (ACC)** calculation is based on the difference between two consecutive frames ($l_{i})$ of the hip angle values ($y_{i})$ and the knee angle values ($x_{i})$. It is obtained by the following steps:

1. Calculating the angular direction of the line segment $l_{i,i+1}$, between the difference of two consecutive points:

| $l_{i,i+1}=\sqrt{{{(x}_{i}-x_{i+1})}^{2}+{{(y}_{i}-y_{i+1})}^{2}}$ | (4) |
| --- | --- |

1. Finding the sine and cosine for the hip (Equation 5) and knee (Equation 6):

| ${sin\theta}_{i,i+1}=\frac{y_{i,i+1}}{l_{i,i+1}}$ | (5) |
| --- | --- |
| ${cos\theta}_{i,i+1}=\frac{x_{i,i+1}}{l_{i,i+1}}$ | (6) |

1. Repeating Equations 5 and 6 for all consecutive points within the cycle, then determining the mean vector length for that point-to-point interval ($a_{i,i+1}$):

| $a_{i,i+1}=\sqrt{{({cos\bar{\theta}}_{i,i+1})}^{2}+ {({sin\bar{\theta}}_{i,i+1})}^{2}}$ | (7) |
| --- | --- |

1. Taking the arithmetic average of all mean vector lengths and thus getting the value of ACC:

| $ACC= \frac{(a_{i,i+1}+ a_{i+1,i+2}\ldots a_{n,i+n})}{n}$ | (8) |
| --- | --- |

The ACC was calculated for each participant and each walking speed first per side, then both sides were averaged. For more detailed information regarding ACC calculation, see[44] and Additional material (Fig. A7).


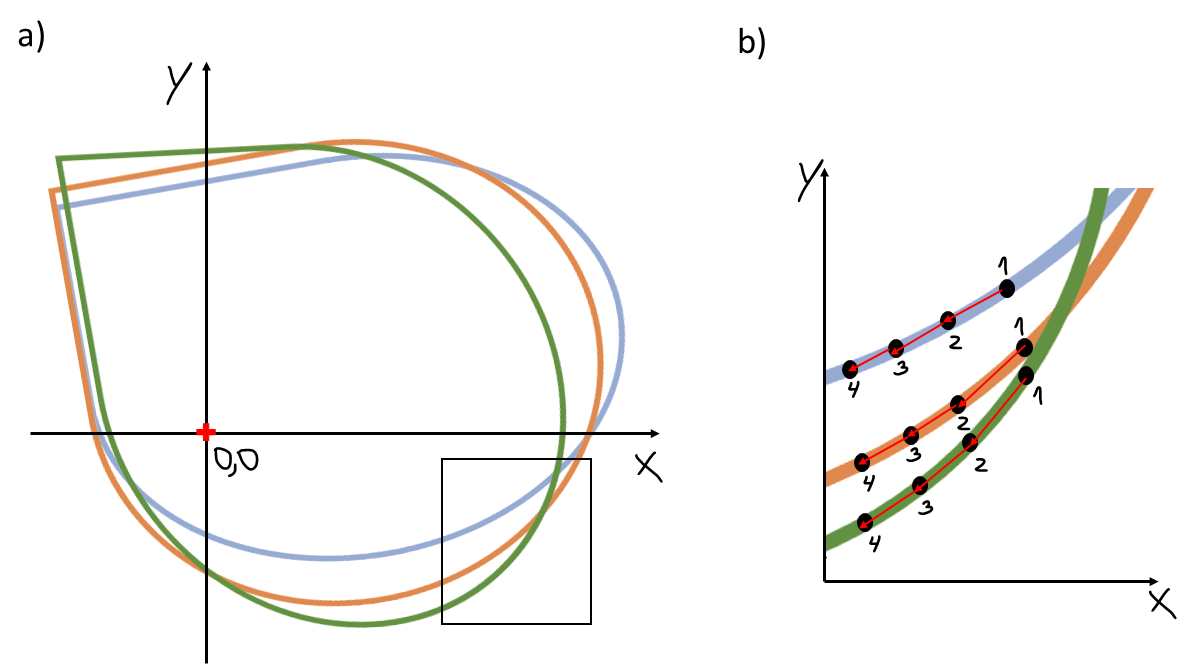


Figure A7. Example cyclograms (plot a) and zoomed in part (plot b) showing line segments (red arrows) between two consecutive points (black dots). The numbers 1-4 indicate arbitrary sample numbers.

The **sum of squared distances (SSD)** is calculated after finding the Euclidean distance between two points in 2D space, where $x_{i}$ and $y_{i}$ represent the hip and knee angles of the first cyclogram, and $x_{j}$ and $y_{j}$ represent the hip and knee angles of the second cyclogram. The calculation for SSD is denoted in Equation 9:

| ${SSD}_{i,i+1}= \sqrt{\sum_{i} {(x_{i}-x_{j})}^{2}+ {(y_{i}-y_{j})}^{2}}$ | (9) |
| --- | --- |

As with ACC, this was first calculated for each participant and each walking speed per side, then both sides were averaged to give the SSD value used for further analysis. Group SSD were obtained by averaging the SSD values for all participants per walking speed.

The **ratio of minimum points to ratio of maximum points** were calculated using the following equations:

| $min\left( magnitude \right)=min(knee+hip)$ | (10) |
| --- | --- |
| $max\left( magnitude \right)=max(knee+hip)$ | (11) |

Where $min \left( magnitude \right)$ denotes the minimum magnitude (lowest point in the cyclogram), and $min(knee)$ and $min(hip)$ are the corresponding knee and hip angles. Similarly, $max \left( magnitude \right)$ (highest point in the cyclogram) is the maximum magnitude and $max(knee)$ and $max(hip)$ are the corresponding knee and hip angles. Note, that the minimum and maximum knee and hip angles do not necessarily correspond to the absolute minimum and maximum knee and hip angles of the cycle; instead, those angles are taken where the magnitude (Equations 10 and 11) is the smallest for minimum and biggest for maximum point calculation. The ratios were obtained using the following equations:

| $ratio(min)=\frac{speed1(min)}{speed2(min)}$ | (12) |
| --- | --- |

| $ratio(max)=\frac{speed1(max)}{speed2(max)}$ | (13) |
| --- | --- |

Where $speed1$ and $speed2$ are two different walking speeds and $\left( min \right)$ and $(max)$ are the minimums and maximums obtained from the cyclograms. The ratios were obtained by comparing the walking speeds in pair-wise, namely fast versus preferred, fast versus slow, and preferred versus slow.
